# Supplementary material for: The interactome of intact mitochondria by cross-linking mass spectrometry provides evidence for coexisting respiratory supercomplexes
Source: Mol Cell Proteomics. 2017 Dec 8;17(2):216–32. doi: 10.1074/mcp.RA117.000470 (PMC5795388; doi:10.1074/mcp.RA117.000470)
Supplement: Supplemental Data [file supp_RA117.000470_133922_0_supp_23978_fzffwf.zip › spectra_annotation/mito_spectra_annotation/242-1-3-1-12-1.pdf]

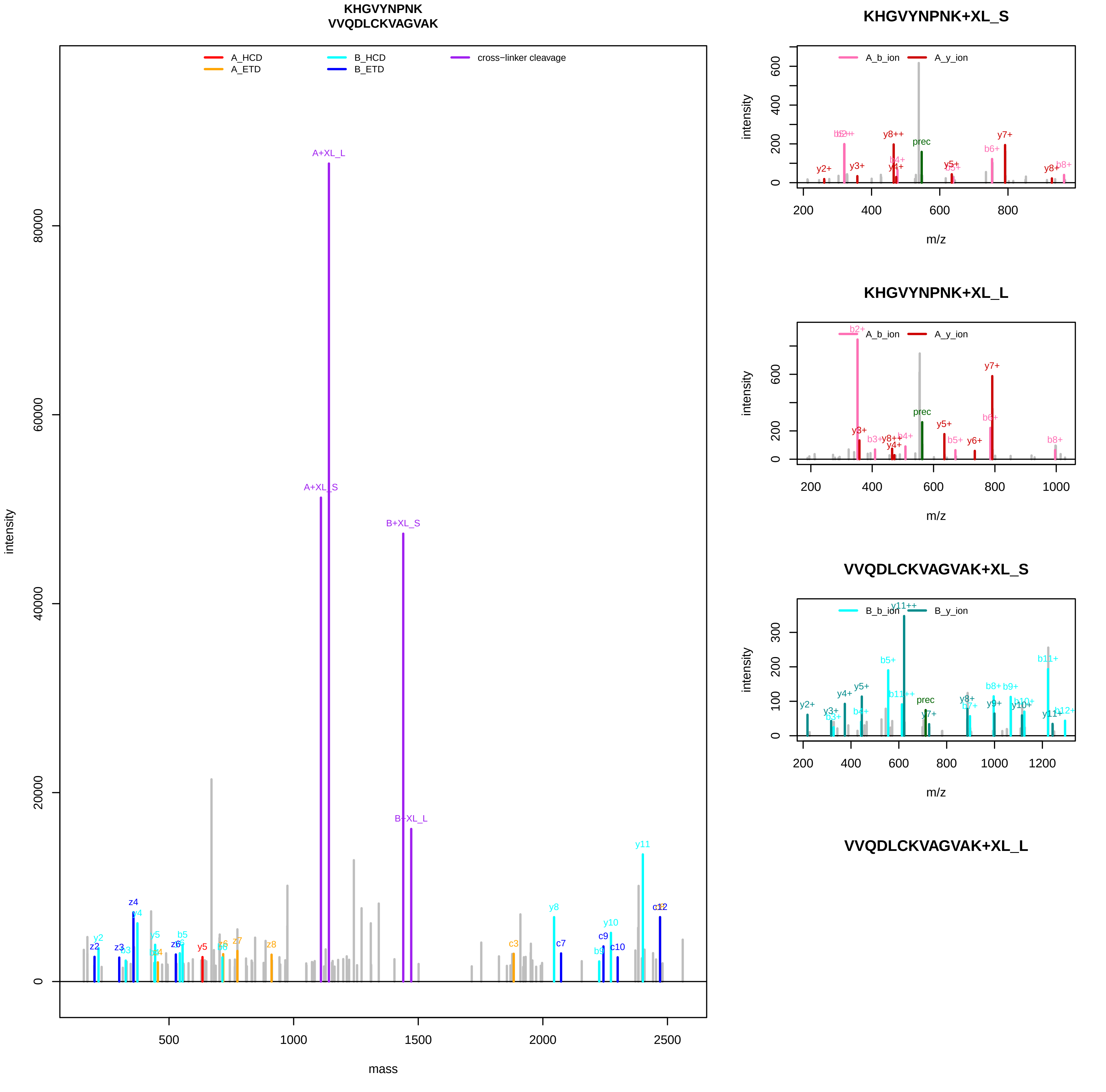
KHGVYNPNK
VVQDLCKVAGVAK
A_HCD
A_ETD
B_HCD
B_ETD
cross−linker cleavage
A+XL_L
A+XL_S
B+XL_S
B+XL_L
y11
y8
c12
y10
c9
c10
c7
c3
b9
z2
y2
z3
b3
z4
y4
y5
b5
z6
b4
z6
b6
z7
z8
y5
intensity
mass
0
20000
40000
60000
80000
500
1000
1500
2000
2500
KHGVYNPNK+XL_S
A_b_ion
A_y_ion
y8++
prec
y7+
b6+
y2+
y3+
b4+
y4+
y5+
y8+
b8+
m/z
KHGVYNPNK+XL_L
b2+
y7+
prec
y3+
b3+
y8++
b4+
y4+
y5+
b5+
y6+
b6+
b8+
VVQDLCKVAGVAK+XL_S
B_b_ion
B_y_ion
y11++
b5+
y5+
y4+
y2+
y3+
b3+
b4+
b11++
prec
y7+
y8+
b7+
b8+
b9+
y9+
b10+
y10+
b11+
y11+
b12+
VVQDLCKVAGVAK+XL_L
